# Ubiquitin-proteasome genes as targets for modulation of cisplatin sensitivity in fission yeast

**DOI:** 10.1186/1471-2164-12-44

**Published:** 2011-01-19

**Authors:** Laura Gatti, Kwang L Hoe, Jacqueline Hayles, Sabina C Righetti, Nives Carenini, Laura Dal Bo, Dong U Kim, Han O Park, Paola Perego

**Affiliations:** 1 Fondazione IRCCS, Istituto Nazionale per lo Studio e la Cura dei Tumori, 20133 Milan, Italy; 2 Functional Genomics Research Center, Korea Research Institute of Bioscience and Biotechnology (KRIBB), Daejeon, Republic of Korea; 3 Cell Cycle Laboratory, Cancer Research UK, London Research Institute, London WC21 3PX, UK; 4 Bioneer Corporation, Daejeon, 306-220, Republic of Korea

## Abstract

**Background:**

The ubiquitin(Ub)-proteasome pathway is implicated in the regulation of a variety of cellular functions and plays a major role in stress response in eukaryotic cells, by targeting misfolded and damaged proteins for degradation. In addition, in the presence of DNA damage, the Ub-proteasome system regulates proteins involved in sensing, repairing, and/or tolerating the damage. Antitumor agents such as cisplatin can activate the pathway, but the role of specific pathway components in cell sensitivity/response to the drug is not known. Since platinum compounds represent clinically relevant antitumor agents and a major limitation to their use is the development of drug resistance, there is an urgent need for identifying targets for improving their efficacy.

**Results:**

In the present study, we performed a genome-wide screening for sensitivity to cisplatin using non-essential haploid deletion mutants of the fission yeast *Schizosaccharomyces pombe*, belonging to a collection of haploid strains constructed through homologous recombination. Using this approach, we identified three Ub-proteasome mutants exhibiting hypersensitivity to cisplatin (*ubp16*, *ubc13 *and *pmt3*) and ten mutants (including *ufd2*, *beta7 20S*, *rpt6/let1*) resistant to the drug. In addition, the importance of lub1 gene emerged from the comparison between the present screening and gene expression profile data previously obtained in fission yeast.

**Conclusions:**

The factors identified in the present study allowed us to highlight most finely the close relationship between the Ub-proteasome system and DNA damage response mechanisms, thus establishing a comprehensive framework of regulators likely relevant also in higher eukaryotes. Our results provide the proof of principle of the involvement of specific genes modulated by cisplatin treatment in cell response to the drug, suggesting their potential role as targets for modulating cisplatin sensitivity. In this regard, the prospective identification of novel targets for modulation of cisplatin sensitivity in an eukaryotic model organism appears particularly intriguing towards the discovery of strategies to overcome cisplatin resistance in human tumors.

## Background

Cisplatin (cis-diammine-dichloroplatinum) is an effective antitumor agent widely used for the treatment of different tumor types [1]. In spite of the efficacy, the curative potential of such an antitumor drug is limited by the occurrence of resistance [2]. Most information about genetic alterations and cellular mechanisms contributing to drug response/resistance comes from mammalian cell systems [1]. Several mechanisms of resistance to cisplatin have been described including reduced drug accumulation, enhanced repair and increased expression of defence factors [2,3]. Some lines of evidence support the concept that altered expression of subsets of genes may be important in determining the sensitivity/resistance to antitumor agents including cisplatin [3]. Given the powerful molecular tools now available, the combination of molecular pharmacology and molecular biology approaches in studying model organisms could lead to a rapid progress in the discovery of strategies to overcome drug resistance. The ease by which yeast can be manipulated together with similarities of yeast cells to cells of more complex metazoans makes many yeast species, (e.g., the fission yeast *Schizosaccharomyces pombe*) very attractive models for the investigation of conserved evolutionary processes occurring in eukaryotes [4]. Using DNA microarrays, we previously found that in fission yeast cisplatin activates a stress response involving various gene groups [3]. In particular, among the transcripts up-regulated by cisplatin in the sensitive strain, several genes belonging to the ubiquitin (Ub)-proteasome pathway were identified [3].

The Ub-proteasome pathway is implicated in the regulation of a variety of cellular functions and plays a major role in stress response. In fact, by degrading misfolded and damaged proteins, the pathway controls processes including cell cycle, cell death and DNA repair [5,6]. The proteasome recognizes ubiquitinated substrates through its Ub receptors and digest them into peptides and free Ubs. The pathway includes Ub activating enzymes (E1), Ub conjugating enzymes (E2) and Ub ligases (E3), all acting in concert to tag substrates with Ub chains. Proteins may be monoubiquitinated or the Ub monomer may act as a point of attachment for additional Ub monomers, resulting in polyubiquitination. The specific biological signal mediated by a polyubiquitin chain is determined, in part, by the chain topology, which is assigned by the Ub lysine residue used for chain extension. Lys48-linked chains have been implicated in targeting proteins for proteasomal degradation, whereas Lys63-linked chains seem to regulate proteins involved in a wide range of processes, including DNA repair, mRNA translation and endocytosis. Indeed, the Ub-proteasome system is known to regulate the transcription of many genes via both proteolytic and non-proteolytic activities [7]. Ub modification of proteins is reversible as Ub may be removed from proteins by de-ubiquitinating enzymes which hydrolyze the isopeptide bond between Ub and the substrate proteins, or by Ub proteases which remove Ub monomers from a polyubiquitin chain.

Since conclusive findings about the specific contribution of different pathways to cisplatin response in fission yeast have been limited by the analysis of small sets of mutants, in the present study we used a large panel of strains to clarify the contribution of single proteasome genes to cisplatin response. In particular, we employed non-essential haploid deletion mutants, belonging to a collection of haploid strains - constructed through homologous recombination in *S. pombe *[8] - to examine sensitivity to cisplatin. Here, we describe our results aimed at clarifying the involvement of specific genes modulated by cisplatin treatment in cell response to the drug. Understanding the relevant genetic/biochemical alterations of the cisplatin response pathway may provide a rational basis for improving therapy in resistant tumors.

## Results and Discussion

### Experimental model

A set of mutants from a genome-wide library containing 2539 haploid strains was used in the present study. The library represents around 70% of non-essential *S. pombe *genes (25% of the genes are considered essential) and around 2% of them belong to the Ub-proteasome pathway (see additional file 1: Figure S1). Using terms from the Gene Ontology (GO) Consortium, each mutant can be assigned at least to one GO annotation (Table 1). The GO project - **The Gene Ontology **http://geneontology.org - is a major collaborative bioinformatics initiative that aims at standardizing the representation of gene and gene product attributes across species. Fission yeast has at least one GO annotation for 98.3% of its known and predicted protein coding genes (corresponding to 5031 genes, as reported in the most recent update -22/02/2010 - of **The *S. pombe *Genome Project **http://www.sanger.ac.uk/Projects), greater than the current percentage coverage for any other organism [9,10]. The GO terms that are most enriched for Ub-proteasome genes are reported in Table 1. They represent approximately 3% of gene products annotated to "biological processes" for fission yeast. See additional file 2: Figure S2 and additional file 3: Figure S3, for tree views from GO.

**Table 1:** Gene Ontology analysis for ubiquitin-dependent processes

		Annotated gene products	
**Name**	**GO terms**	***S. pombe***	**All organisms**

All gene products	All	5230	463.707

Biological processes	GO:0008150	5219	349,949

Ub-dependent protein catabolic processes	GO:0006511	177	2393

Proteasomal-Ub dependent protein catabolic processes	GO:0043161	110	733

Protein ubiquitination	GO:0016567	124	1865

Protein modification by small protein conjugation or removal	GO:0070467	162	1734

The screening of the library was performed in liquid culture assays, because this test is more suitable than tests on plates to examine the effect of cisplatin, which by virtue of its chemical features (i.e., electrophilicity) easily reacts with the abundant nucleophilic components of yeast extract plates (e.g., organic sulphur: methionine, cysteine), thereby becoming inactive. In preliminary experiments, the optimal drug concentrations to employ in the deletion mutant screening were determined using the wild-type 972 h^- ^and mutant *rad3 *strain because *rad3 *is hypersensitive to cisplatin (IC_50 _= 0.01 ± 0.009 μM) and 972 h^- ^is the strain from which *rad3 *mutant was generated.

### Sensitivity of *S. pombe *deletion mutants to cisplatin

**Figure 1:**
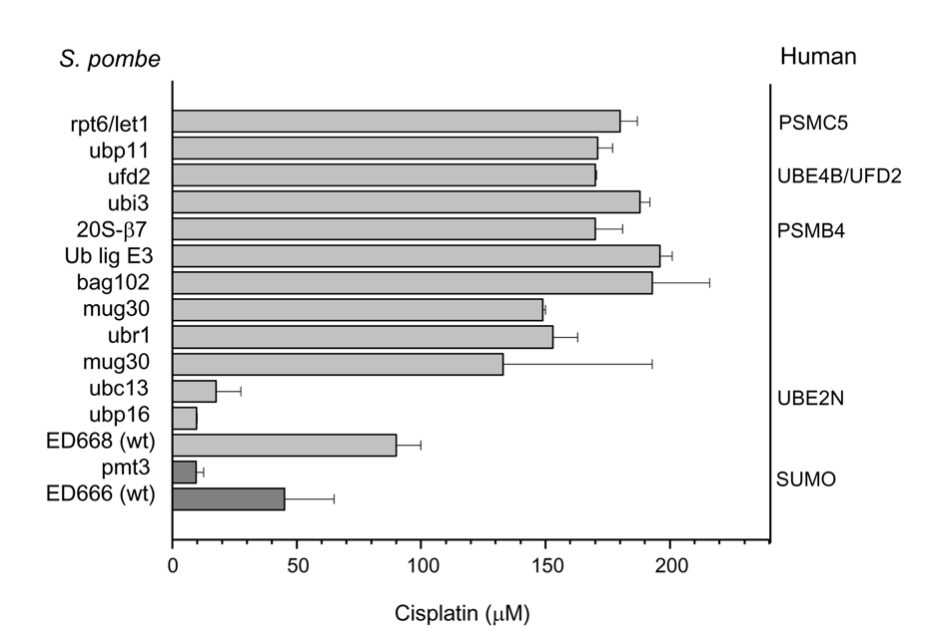
**Sensitivity of *S. pombe *deletion mutants to cisplatin**. In the bar-chart are reported three cisplatin-sensitive and ten cisplatin-resistant strains with the corresponding human homolog/ortholog genes, when described. IC_50 _values are the means ± standard deviation of at least three independent experiments. WT, wild-type strains (ED 668: h^+ ^*ade6-M216/ura4-D18/leu1-32 *and ED666: h^+ ^*ade6-M210/ura4-D18/leu1-32*, respectively).

When assaying the cisplatin sensitivity of 47 deletion mutants belonging to the proteasome pathway, we identified a number of cisplatin sensitive and resistant mutants in comparison to the corresponding wild-type strains (Table 2). A list of the *S. cerevisiae *and human homologous/horthologous genes corresponding to those evaluated for cisplatin sensitivity is reported in Table 3. In particular, we found that 3 deletion mutants were cisplatin-sensitive (*ubp16*, *ubc13 *and *pmt3*) and that 10 mutants were cisplatin-resistant (Figure 1).

**Table 2:** Cisplatin sensitivity of Ub-proteasome pathway deletion mutants

Gene ID	Annotation	IC_50 _(mM) ± SD	Cisplatin response
Mutant strains^a^			

SPAC26A3.16	dph1	0.068 ± 0.023	

SPAC328.06	ubp2	0.079 ± 0.06	

SPAC24H6.03	pcu3	0.053 ± 0.006	

SPAC167.07c	cul3	0.048 ± 0.03	

SPAC12B10.01c	UB-lig (E3)	0.133 ± 0.06	R

SPBC6B1.06c	ucp2, ubp14	0.08 ± 0.02	

SPCC1682.12c	ubp16	0.0098 ± 0.0002	HS

SPBP8B7.21	ubp3	0.11 ± 0.08	

SPBC19C7.02	ubr1	0.153 ± 0.01	R

SPBP8B7.27	mug30	0.149 ± 0.001	R

SPCC1442.07c	UB metalloprotease	0.061 ± 0.006	

SPAC27F1.03c	uch1	0.066 ± 0.018	

SPAC31G5.18c	UB family...	0.025 ± 0.05	

SPCC188.08c	ubp22/ubp15	0.065 ± 0.011	

SPAC11E3.04c	ubc13/spu13	0.0175 ± 0.01	HS

SPAC10F6.07c	ubc6	0.062 ± 0.011	

SPAC1250.03	ubc14	0.069 ± 0.012	

SPAC13A11.04c	ubp8	0.044 ± 0.003	

SPBC800.12c	UB family...	0.057 ± 0.006	

SPBC106.16	pre6	0.053 ± 0.01	

SPBC409.06	uch2	0.052 ± 0.02	

SPBC530.03c	bag102/bag1-b	0.193 ± 0.023	R

SPBC16G5.03	UB-lig (E3)	0.051 ± 0.013	

SPBC19G7.09	ulp1(SUMO-deconj)	0.054 ± 0.016	

SPCC1919.15	brl1	0.052 ± 0.019	

SPBC2A9.04c	UB-lig (E3)	0.196 ± 0.005	R

SPBC577.10	20S (sub.β7)	0.170 ± 0.011	R

SPAC17G8.10c	dma1	0.063 ± 0.004	

SPAC6G10.11c	ubi3	0.188 ± 0.004	R

SPAC20H4.10	ufd2	0.17 ± 0.0006	R

SPBC19C2.04c	ubp11	0.171 ± 0.006	R

SPBC15C4.06c	UB-lig (E3)	0.055 ± 0.006	

SPAC23G3.08c	ubp7	0.06 ± 0.004	

SPBC23G7.12c	let1/rpt6 (19S)	0.18 ± 0.007	R

SPBC887.04c	lub1	0.054 ± 0.005	

SPAC15A10.11	ubr11	0.062 ± 0.019	

SPAC1198.09	ubc16	0.032 ± 0.013	

SPAC17C9.13c	cut8	0.028 ± 0.002	

SPBC18H10.08c	ubp4	0.054 ± 0.019	

SPCC790.02	pep3	0.024 ± 0.003	

SPBC16E9.11c	pub3	0.059 ± 0.005	

SPAC1805.15c	pub2	0.062 ± 0.005	

SPCC1682.16	rpt4	0.03 ± 0.02	

Wild-type strain^a^			

ED668: h^+ ^*ade6-M216/ura4-D18/leul-32*		0.09 ± 0.01	

			

Mutant strains^a^			

SPAC6G9.08	ubp6	0.052 ± 0.008	

SPBC6B1.05c	atg7	0.073 ± 0.009	

SPBC2D10.20	ubc1	0.055 ± 0.015	

SPBC365.06	pmt3	0.0096 ± 0.003	HS

			

Wild-type strain^a^			

ED666: h^+ ^*ade6-M210/ura4-D18/leul-32*		0.045 ± 0.02	

			

Cisplatin sensitive strain^a^			

SPBC216.05	rad3	0.01 ± 0.009	

^a ^Sensitivity was evaluated using a microtiter growth inhibition assay with a 48 h drug exposure. IC_50 _values are the means ± standard deviation of at least three independent experiments. The IC_50 _was defined as the drug concentration that reduced the absorbance to 50% of the value measured for the untreated control culture, and was compared to cisplatin sensitivity of the corresponding wild-type parental strain. Hypersensitive strains are indicated as HS and resistant strains as R.

**Table 3:** Ub-proteasome pathway deletion mutants evaluated for cisplatin sensitivity: *S. cerevisiae *and human homolog/ortholog genes

	Homolog/Ortholog	
**Annotation**	***S. cerevisiae***	**Human**

dph1	DSK2	-

ubp2	UBP2	-

pcu3	-	-

cul3	UFD4	-

UB-lig (E3)	-	-

ucp2, ubp14	UBP14	-

ubp16	UBP10	-

ubp3	YER151C	-

ubr1	UBR1/PTR1/UBR2	-

mug30	HUL4	-

UB-metalloprotease	-	-

uch1	YUH1	UCHL3

UB family...	-	-

ubp22/ubp15	UPB15	USP7

ubc13/spu13	UBC13	UBC13/UBE2N

ubc6	UBC6	UBE2J2

ubc14	-	-

ubp8	UBP8	USP22

Ub family...	-	-

pre6	PRE6	PSMA8

uch2	YUH1	UCHL3

bag102 / bag1-b	SNL1	-

UB-lig (E3)	-	-

ulp1 (SUMO-deconj)	ULP1	SENP8

brl1	BRE1	-

UB-lig (E3)	SAN1	-

20S (sub.β7)	PRE4	PSMB4

dma1	DMA1	-

ubi3	RPS31	-

ufd2	UFD2	UBE4B

ubp11	-	-

UB-lig (E3)	-	-

ubp7	UBP7	USP45

let1/rpt6 (19S)	RPT6	PSMC5

lub1	DOA1	PLAA

ubr11	-	UBR2

ubc16	-	-

cut8	-	-

ubp4	DOA4/UBP4	-

pep3	-	VPS18

pub3	RSP5	NEDD4

pub2	RSP5	NEDD4

rpt4	RPT4	PSMC6

ubp6	UBP6	USP14

atg7	ATG7	ATG7

ubc1	UBC1	UBE2K

pmt3	SMT3	SUMO

### Cisplatin-sensitive deletion mutants

**Ubp16 **(SPCC1682.12c), similar to *S. cerevisiae *UBP10 (YNL186W), is a Ub-specific processing protease endowed with Ub-C-terminal hydrolase activity, and is localized to the nucleolus of *S. pombe*. The corresponding budding yeast homolog gene UBP10 encodes a deubiquitinating enzyme whose loss of function results in a complex phenotype displaying perturbations in different cellular processes, characterized by slow growth rate, partial impairment of silencing at telomeres, reduced subtelomeric repression and up-regulation of stress-responsive genes. This complex phenotype is also accompanied by accumulation of reactive oxygen species and by appearance of apoptosis-like phenotypical markers [11]. UBP10 is directly involved in the maintenance of histone H2B ubiquitination levels, that is critical for the transcriptional and cell cycle response to DNA damage [12]. Such observations are particularly interesting since the major epigenetic mechanisms controlling histone modifications and nucleosome remodelling are extremely well conserved between yeast and higher organisms [13]. Consequently, UBP10 inactivation induced a transcriptional oxidative stress response accompanied by a subpopulation of apoptotic cells which accumulated reactive oxygen species [14,15]. The corresponding human homolog gene has not been yet described. Although significant progress has been made in the characterization of enzymes that ligate Ub to target proteins in humans, little is known about the removal of Ub from Ub-conjugates. Yet, the activity of Ub-specific proteases (USPs) is likely to be central to the regulation of all processes in which Ub is involved, both removing Ub to rescue from degradation or by removing residual Ub to assist in proteasomal degradation. The human genome encodes 60-70 predicted members of the USP family, and at least five major classes have been identified, one of which gathers Ub-processing proteases (UBP) including UBP10. Collectively, several findings identify USPs as important regulators of biological processes and potential targets for the treatment of human tumors [16-19].

**Ubc13 **(SPAC11E3.04c), is a Ub-conjugating enzyme (E2), involved in protein ubiquitination, DNA repair, DNA post-replication repair (PRR) and in targeting of Lys63 histone, similarly to the *S. cerevisiae *homolog gene YDR092W (UBC13). In fission yeast, deletion of Ubc13 results in an increased sensitivity to DNA damaging agents, i.e., the alkyating agent methylmethanesulfonate (MMS) and UV radiation [20]. Since the ubiquitination of PCNA plays a crucial role in regulating replication past DNA damage, this aspect was investigated also in *S. pombe *[21]. In particular, it has been shown that the genetic requirements for mono- and polyubiquitination of PCNA are similar to those in *S. cerevisiae*, namely that monoubiquitination requires Rhp18^Rad18^, whereas polyubiquitination requires Rad8^Rad5^, Ubc13 and Mms2 [21]. DNA PRR is a tolerance mechanism that allows cells to survive DNA damage that is unrepaired or unrepairable. PRR includes translesion DNA synthesis that is "error-prone" and a second activity that is largely "error-free". In budding yeast, the UBC13 gene codes for an Ub-conjugating enzyme involved in the error-free DNA PRR pathway. After DNA damage, Ubc13p interacts with Mms2p to assemble Ub chains at the Ub-Lys63 residue of PCNA, instead of the conventional Lys48 residue that is the main signal to target a substrate for proteolysis by 26S proteasome. The involvement of UBC13 in cellular tolerance to DNA damage is further supported by its inducibility in response to treatment with DNA-damaging agents such as MMS and UV radiation [22,23]. The human homolog of *S. pombe *Ubc13, is UBE2N/UBC13, a Ub-conjugating enzyme requiring the presence of a Ubc variant (Uev) for poly-ubiquitination. In particular, divergent activities of mammalian Ubc13 rely on its pairing with either of two Uevs, Uev1A or Mms2 [24,25].

**Pmt3 **(SPBC365.06) gene product is SUMO (small, ubiquitin-like modifier), one of a number of Ub-like protein that are post-translationally covalently attached to one or more Lys residues on target proteins. Although it has only 18% sequence identity with Ub, its structure resembles that of Ub. However, unlike Ub, mammalian SUMO and its budding yeast homologue SMT3 have been shown to be more important for post-translational protein modification than for protein degradation [26]. Indeed, SUMO modification has a variety of cellular functions, including roles in transcription, DNA damage response, cell cycle and nuclear transport. Recently, Pmt3 has been shown to be required for SUMO-targeted Ub ligase-dependent ubiquitination of target proteins [27]. As an example, *S. pombe *PCNA is sumoylated in S phase following DNA damage [21]. The process of sumoylation resembles that of ubiquitination. SUMO is produced as a precursor protein that needs to be cleaved to the mature form by one or more specific SUMO-proteases. Genetic analyses showed that the pmt3 gene is not essential for viability (while SUMO is essential for viability in mammals and *S. cerevisiae*), but it may be essential for the checkpoint coupling mitosis to the completion of DNA replication and the DNA damage response. Deletion mutants for pmt3 were strikingly sensitive to the DNA synthesis inhibitor hydroxyurea (HU), MMS and UV radiation, and the microtubule-destabilizing agent thiabendazole. However, it has been proposed that pmt3 is involved in the DNA damage tolerance process rather than in the checkpoint itself, similarly to rad31 (SUMO activator subunit) and hus5 (SUMO conjugator) [26]. In fission yeast, sumoylation is involved also in chromosome segregation and telomere length maintenance. Loss of pmt3 function caused a striking increase in telomere length [26]. More recently, a role for SUMO chain formation in response to replication arrest in *S. pombe *has been established [27]. In addition, a variable pattern of response to DNA damaging agents has been reported in the budding yeast *SIZ1 *gene mutant, which is characterized by resistance to anthracyclines (doxorubicin, daunorubicin) and sensitivity to cisplatin and camptothecin [28]. Since SIZ1 is an E3-ligase of the SUMO pathway, sumoylation defects may impair drug response.

### Cisplatin-resistant deletion mutants

Although we were mainly interested in identifying drug-sensitive mutants with the final goal to establish novel targets for increasing cisplatin sensitivity, the present study allowed us to identify several cisplatin-resistant strains (Figure 1). Among these, here we describe those carrying deletions in genes whose human homolog/ortholog has been already described (Figure 1).

**Ufd2 **belongs to the Ub-conjugation factor "E4 family" and is involved in N-terminal Ub-fusion degradation (UFD) pathway, required for the degradation of oligo-ubiquitinated substrates [29]. Notably, UFD2 has a crucial activity in *S. cerevisiae *because it binds proteins modified by one or two moieties only, thus harbouring a too short chain for triggering degradation, and is able to catalyze an extension of the multi-Ub chain [30,31]. A two-step reaction, i.e., oligo-ubiquitination followed by E4-catalyzed multi-ubiquitination, could offer a double layer of control, giving the possibility for two consecutive functions [31]. Moreover, UFD2 may have a role in retro-translocation and endoplasmic reticulum-associated degradation (ERAD) pathway, where misfolded or abnormally assembled proteins are targeted for degradation. Importantly, the bulk of UFD2 appears to reside in the nucleus, possibly with bound ubiquitinated substrates (e.g., transcription factors). The mammalian homolog of yeast Ufd2/UFD2 is UFD2a/UBE4B gene, that contains a U-box at its C-terminus and functions as an E3 as well as an E4 Ub-ligase [30]. It has been demonstrated that UFD2a mediates the proteasomal turnover of p73 in a Ub-independent manner and that it might play an important role in the regulation of cisplatin-induced apoptosis mediated by p73 [32]. More recently, it has been suggested that UFD2a might regulate also cisplatin-mediated cell death by p63 [33].

The SPBC577.10 gene codes for the **β7 subunit of 20S **proteasome, whose corresponding ortholog gene in *S. cerevisiae *is PRE4. A mutant strain with defects in PRE4 displays cycloheximide resistance [34]. The corresponding human gene/protein (PSMB4) is evolutionarily conserved and directly interacts with SNEV (senescence evasion factor), a protein with E3 ligase activity, which is also involved in DNA double-strand break repair and splicing, whose deficiency results in apoptosis and decreased cell survival after DNA damage [34,35]. It has been suggested that PSMB4 might be a major site for proteasome regulation, where signals from the outside might be transduced inside to the protease activities [34]. Altered expression of the PSMB4 gene was recently observed in association with various tumor types (glioblastoma, ovarian carcinoma, hepatocarcinoma, prostate carcinoma) through different approaches [36-39]. Interestingly, another human gene coding for the 20S proteasome unit β-type 7 (PSMB7), is associated with anthracycline resistance and is a prognostic biomarker in breast cancer [40].

**Rpt6/Let1 **is one of six ATPases of the 19S regulatory particle of the 26S proteasome involved in the degradation of ubiquitinated substrates; its *S. cerevisiae *homolog gene (Rpt6p) is bound by Ub-protein ligases Ubr1p and Ufd4p and localized mainly to the nucleus throughout the cell cycle. The Rpt6 protein (also known as Sug1 in budding yeast and hSug1/TRIP1/S8/PSMC5 in mammals) has been found to associate with a number of activators and to be localized on some promoters in mammals [41]. In particular, Rpt6 has been localized on p21^WAF1 ^promoter where it interacts with p53 after DNA damage. The knockdown of Rpt6 results in increased occupancy of the p21^WAF1 ^promoter by p53 and increase transcription of the gene [41].

### Modulation of Ub-proteasome genes by cisplatin

We previously studied genome-wide transcriptional profiles in *S. pombe*, demonstrating that cisplatin activates a stress response involving genes belonging to different pathways (glutathione, heat-shock, DNA repair), including Ub-proteasome system) [3]. In such an analysis, the *S. pombe *wild-type sensitive strain 972 h^- ^was exposed to a cytotoxic cisplatin concentration and modulation of gene expression was examined. The group of transcripts at least two-fold up-regulated by cisplatin in this strain comprised a subset of transcripts belonging to the Ub-proteasome pathway (Table 4). Only three of them were found to be included in the present set of non-essential deletion mutants (Lub1, Ubc6 and Uch2). When we tested cisplatin sensitivity of these specific deletion mutants (see Table 2), we obtained IC_50 _values similar to that of the corresponding wild-type parental strain.

**Table 4:** Expression profiling studies of fission yeast with DNA microarrays - ***S. pombe ***Functional Genomics Lab http://www.sanger.ac.uk/PostGenomics/S_pombe/-: modulation of genes belonging to the ubiquitin-proteasome pathway

		Homolog/Ortholog	
**Gene name**	**Annotation**	***S. cerevisiae***	**Human**

Lub1	Ub proteolysis	DOA1/UFD3	PLAA

ubi4	Ub-attachment to target	UBI4	UBC

ubc6	Ub ligase	UBC6	UBE2J2

SPAC22A12.14c	Ub protease	-	-

Ubx3:mug39	CDC48-dependent degradation	UBX3	NSFL1C

Uch2	26S Ub-hydrolase	YUH1	UCHL3

Rpn12:mts3	19S regulatory protein	RPN12/NIN1	PSMD8

Ubx2:ucp13	CDC48-dependent degradation	UBX5	-

Rpn3	19S regulatory protein	RPN3/SUN2	PSMD3

SPCC4G3.13c	Cue1/4 domain	CUE1-4/KIS4	-

^a ^Modulated transcripts are reported for which at least a two-fold up-regulation was found.

^b ^The *S. pombe *wild-type sensitive strain 972 (h^-^, no auxotrophic markers) was exposed to a cisplatin concentration inhibiting colony growth by 80% (1.28 mM) after 4h drug exposure.

Among the induced transcripts, **Lub1 **attracted our attention because a precise and important role in DNA damage response has been recently ascribed to its corresponding budding yeast homolog gene, DOA1/UFD3 [12]. In particular, DOA1 has been shown to help to control the DNA damage response by channelling Ub from the proteasomal degradation pathway into pathways that mediate altered DNA replication and chromatin modification, thus acting in supplying Ub for the DNA damage response. Elements of the DNA damage response that appear to rely on DOA1 include the ubiquitination of both PCNA and histone H2B. Indeed, DOA1 interacts with other factors involved in producing or maintaining ubiquitinated both PCNA and H2B, i.e., UBC13 and UBP10 (the *S. cerevisiae *homolog of fission yeast Ubc13 and Ubp16, see above for details). Thus, such an observation suggests a link between three different factors belonging to the Ub-proteasome pathway identified in *S. pombe *with two different approaches (global gene expression analysis and cell sensitivity screening), and possibly involved in cellular response to cisplatin. Moreover, the lack of cisplatin-hypersensitivity observed in our *Lub1 *deletion mutant (see Table 2), may reflect the presence of redundant factors as suggested by Lis and Romesberg [12]. Indeed, in budding yeast *doa1*Δ and *ubi4*Δ mutants share several phenotypes including sensitivity to heat, canavanine and other DNA-damaging agents (HU, MMS) [42]. In contrast, the budding yeast *UBI4 *deletion mutant displays resistance to cisplatin together with other mutants of the proteasome pathway including *BUL1, UBP13, UFD4 and UMP1 *[43]. Both UBI4 (which has conventionally been thought to be the major source of Ub for various stress responses) and DOA1 might supply Ub for the DNA damage response. Similarly, in fission yeast the corresponding UBI4 homolog gene (*S. pombe *Ubi4, SPBC337.08c) may replace Lub1 absence. Accordingly, Ubi4 gene expression resulted up-regulated by cisplatin in our previous study, similarly to Lub1 (see Table 4).

As reported in Table 4, the human ortholog of *S. pombe *Lub1 and *S. cerevisiae *DOA1/UFD3, is phospholipase A2 (PLA2)-activating protein (PLAA, also known as PLAP), that has been implicated in a variety of biological processes that involve the Ub system [44]. In particular, it has been linked to the maintenance of Ub levels, but the mechanism by which it accomplishes this is unclear [44]. Interestingly, it has been recently demonstrated that human PLAA enhances cisplatin-induced apoptosis in HeLa cells [45]. Transcriptional induction of PLAA by cisplatin can potentially promote cytotoxicity through phospholipase A2 activation and arachidonic acid accumulation. Interestingly, carboplatin-sensitive cells from ovarian cancer patients expressed higher levels of PLAA than their resistant counterparts [46]. The C-terminal domain of PLAA binds p97/Cdc48, an AAA ATPase which, among other functions, helps in transferring ubiquitinated proteins to the proteasome for degradation [44]. In addition, PLAA is also associated with HDAC6, a unique cytoplasmic deacetylase capable of interacting with Ub and a master regulator of the cell protective response to cytotoxic protein aggregate formation [47,48].

## Conclusions

To maintain the genome, cells have evolved multiple pathways to detect and respond to DNA damage. The cellular response to DNA damage has been particularly well characterized in the fission yeast *S. pombe*. An important way in which various organisms coordinate facets of the DNA damage response is the post-translational modification of proteins. While phosphorylation has received a great deal of attention, it has become increasingly clear that other types of post-translational modifications, such as ubiquitination, also play critical roles. Ub is an essential modifier conserved in all eukaryotes from yeast to human and existing in several cellular compartments. During normal growth, a significant portion of Ub is used to target proteins for proteasomal degradation, and it is presumably sequestered within these pathways. However, in the presence of DNA damage, Ub must be quickly made available for post-translational modification of proteins involved in sensing, repairing, and/or tolerating the damage (such as PCNA and histone H2B). The present study supports that specific proteasome genes can contribute differently to cisplatin response. Only a few of yeast genes appear to regulate sensitivity *per se *suggesting pathway redundancy. The prospective identification of novel targets for modulation of cisplatin sensitivity in an eukaryotic model organism appeared particularly intriguing towards the discovery of strategies to overcome cisplatin resistance in human tumors. In principle, a variety of approaches may be employed in an attempt to sensitize cancer cells to cisplatin (e.g., pharmacological inhibitors, small interfering RNAs, antisense oligonuclotides). In the context of the Ub-proteasome pathway, the development of small molecules is still at an early stage, but some research groups are already looking at attacking components of the Ub-proteasome pathway [49]. In this regard, the definition of the interactome of the specific enzymes is crucial to select the most promising druggable targets. Also, a major effort is required to better understand pathway redundancy because, although ubiquitin ligases have shown a high degree of substrate specificity, their inhibition may be counteracted by the activation of alternative pathway components critical for cell survival maintenance. The level of complexity of the Ub-proteasome pathway is high as also deubiquitinases can be regarded as druggable targets (e.g., USP7). Moreover, the pairing of the pathway components with different substrates may result in divergent activities [24,25].

**Figure 2:**
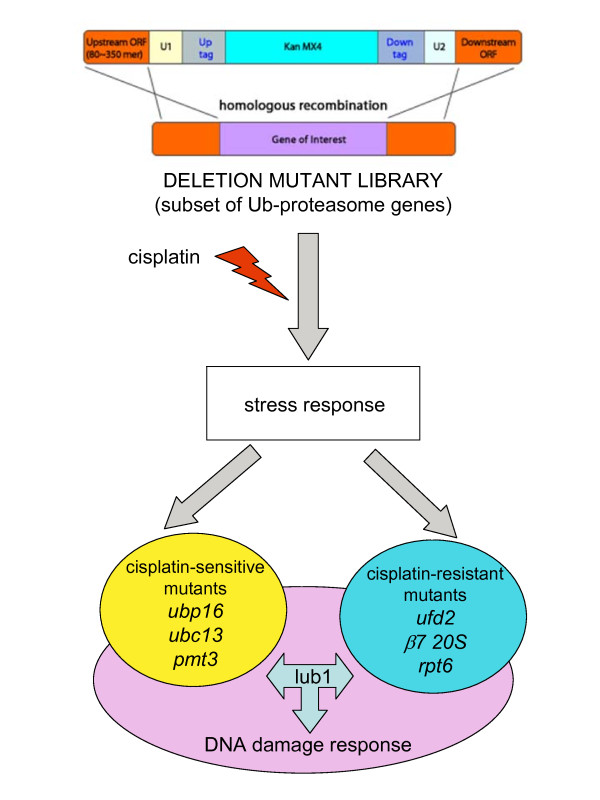
**Summary of the relevant findings of the study**. The experimental approach used here and the obtained results are illustrated to point out the fact that the Ub-proteasome genes identified in the present study as regulators of cisplatin response are all implicated in controlling DNA damage response.

In the present study, we identified three Ub-proteasome mutants exhibiting hypersensitivity to cisplatin, i.e., *Ubp16*, *Ubc13 *and *Pmt3*. Although with very distinct functions, the proteins encoded by those genes play critical roles for DNA damage response, thereby representing attractive targets to investigate possible mechanisms of cisplatin resistance in human tumor cell systems (Figure 2). With respect to factors whose loss confers cisplatin resistance, Ufd2 might play a role in cisplatin-induced apoptosis [32,33]. Our screening also highlighted the importance of the β7 subunit of 20S proteasome, whose corresponding human ortholog gene is PSMB4. Since PSMB4 is implicated in proteasomal degradation of SNEV, the absence of PSMB4 may produce resistance as a consequence of increased survival favoured by SNEV [34,35].

To the best of our knowledge, none of the budding yeast homologues of the fission yeast mutants described in the present study has been previously linked to cisplatin response.

When we compared the present screening results with those obtained in previous global gene expression study, the importance of Lub1 emerged. Indeed, the corresponding budding yeast ortholog gene (DOA1/UFD3) has a precise and important role in DNA damage response and appears to regulate the ubiquitination of both PCNA and histone H2B, through the interaction with UBC13 (Ubc13 of *S. pombe*) and UBP10 (Ubp16 of *S. pombe*). PCNA is at the very heart of many essential cellular processes, such as DNA replication, repair of DNA damage, chromatin structure maintenance, chromosome segregation and cell-cycle progression. This puts PCNA in a central position in determining the fate of replication fork, which ultimately determines both tumor progression as well as the outcome of anticancer treatment [50]. In addition, recent advances have defined a clear role for histone H2B ubiquitination in transcriptional regulation and the enzymes regulating this post-translational modification have been linked to tumorigenesis [51].

In summary, we can conclude that the cell sensitivity screening shown in the present study, together with evidences resulting from previous *S. pombe *gene expression analysis, uncover novel putative targets for modulation of cisplatin sensitivity, particularly intriguing towards the discovery of strategies to overcome cisplatin in human tumors.

## Methods

### ***S. pombe*****deletion library**

A genome-wide deletion mutant library was constructed in large scale by PCR-based targeted mutagenesis at each target ORF, on the base of the *S. pombe *genome sequence information provided by the public website at **The Wellcome Trust Sanger Institute **http://www.sanger.ac.uk. The details of library construction have been recently reported by Kim et al. [8]. Briefly, the deletion cassettes were transformed into *S. pombe *SP286 diploid host strain (h^+^/h^+^, *ade6-M210/ade6-M216 ura4-D18/ura4-D18 leu1-32/leu1-32*) and deletion mutants were screened by G418 selection, see also **Bioneer *Schizosaccharomyces pombe ***http://pombe.bioneer.co.kr for details. Non-essential haploid mutants were obtained from diploid mutants [8]. The library was provided by the Bioneer Corporation and the Korea Research Institute of Biotechnology and Bioscience [8,52]. All viable mutants were screened.

### Culture conditions, cytotoxicity assays and drugs

**Figure 3:**
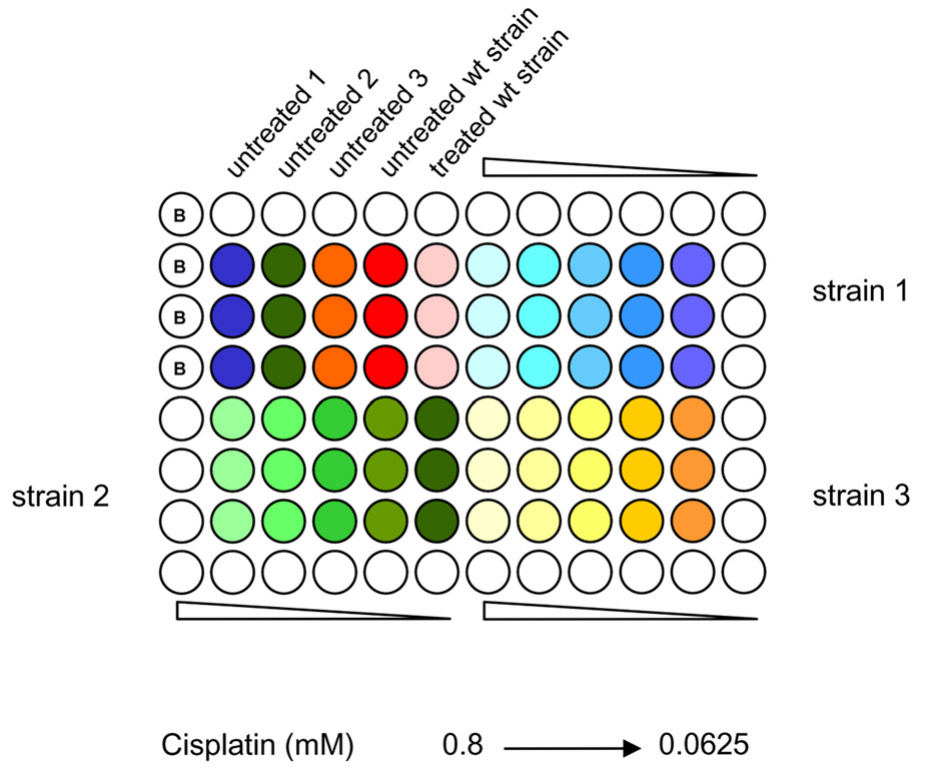
**Cell sensitivity assay**. Layout of the standard assay used for screening cell sensitivity of deletion mutants. A growth inhibition assay performed in 96-well microtiter plates was used. After 48 h drug exposure the absorbance at 595 nm was measured.

Yeast cultures were grown at 30°C in YE+AUL medium (5 g of yeast extract, 30 g of glucose, 0.075 g of adenine, 0.075 g of uracil, 0.25 g of leucine and 0.1 g of G418/liter). A growth inhibition assay performed in microtiter plates was used to evaluate the antiproliferative effect of cisplatin (Figure 3). Preliminary experiments were performed to verify the linearity of the relation between cell number and absorbance at 595 nm. Cell cultures were grown overnight in liquid medium until mid-log phase. Eight thousand cells were then seeded in 96-well microtiter plates and incubated in drug-containing medium. Plates were then incubated for 48 h at 30°C, at which time the absorbance at 595 nm was measured. The IC_50 _was defined as the drug concentration that reduced the absorbance to 50% of the value measured for the untreated control culture, and was compared to cisplatin sensitivity of the corresponding wild-type parental strain (ED 668: h^+ ^*ade6-M216/ura4-D18/leu1-32 *or ED 666: h^+ ^*ade6-M210/ura4-D18/leu1-32*). In each experiment, the mean IC_50 _values obtained for each strain were divided by the mean IC_50 _value of the corresponding wild-type parental strain to evaluate the occurrence of hypersensitivity or resistance. When the obtained ratio was < 0.25 the strain was considered hypersensitive (HS), whereas strains were considered resistant (R) when the ratio was > 1.5. The 972 h^- ^strain and a cisplatin-hypersensitive strain (*rad3*) [53] were used in preliminary experiments to optimize the assay. Each experiment was repeated at least three times using triplicate wells. Cisplatin (Sigma) was freshly dissolved in 0.9% NaCl.

### List of abbreviations

HU: hydroxyurea; MMS: methyl methanesulfonate; GO: Gene Ontology; PLAA: phospholipase A2 (PLA2)-activating protein; PRR: postreplication repair; SUMO: small Ub-like modifier; Ub: ubiquitin; USP: Ub specific proteases.

## Authors' contributions

LG participated in the design of the study, performed data analysis and drafted the manuscript. KLH participated in study design, in discussions and critical reading of the manuscript. JH carried out the molecular genetic studies for the construction of the deletion mutants. SCR, NC and LDB performed strain growth and storage as well as chemosensitivity assays. DUK and HOP participated in deletion library construction and design. PP conceived the study, participated in its design and coordination and helped to draft the manuscript. All authors read and approved the final manuscript.

## Supplementary Material

Additional file 1 **Figure S1. Scheme of the deletion haploid mutant library**. The scheme represents the deletion haploid mutant library in terms of percentage of non-essential genes and of Ub-proteasome genes as compared to the whole *S. pombe *genome. Click here for file

Additional file 2 **figure S2**. Tree view from **The Gene Ontology **http://geneontology.org relative to the Ub-proteasome system in *S. pombe*. In particular, the "**proteasomal ubiquitin-dependent protein catabolic process (GO:0043161)**" annotation is shown. The tree structure holds information about GO terms and the relationships between them. Each box in the tree view contains the GO ID and the GO term name. Click here for file

Additional file 3 **figure S3**. Tree view from **The Gene Ontology **http://geneontology.org relative to the Ub-proteasome system in *S. pombe*. In particular, the "**protein modification by small protein conjugation or removal (GO:0070647)**" annotation is shown. The tree structure holds information about GO terms and the relationships between them. Each box in the tree view contains the GO ID and the GO term name. Click here for file
