# Supplementary figures and images for: Ubiquitin-proteasome genes as targets for modulation of cisplatin sensitivity in fission yeast
Source: BMC Genomics. 2011 Jan 19;12:44. doi: 10.1186/1471-2164-12-44 (PMC3032702; doi:10.1186/1471-2164-12-44)

*S. pombe* genome

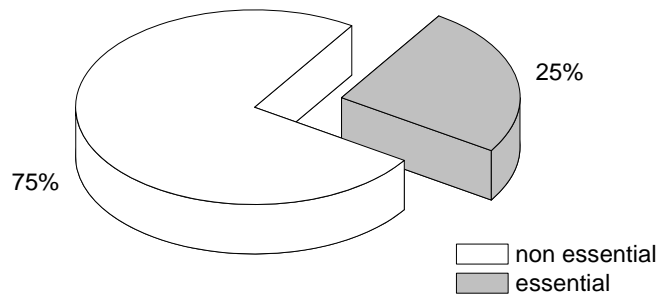

Non-essential genes

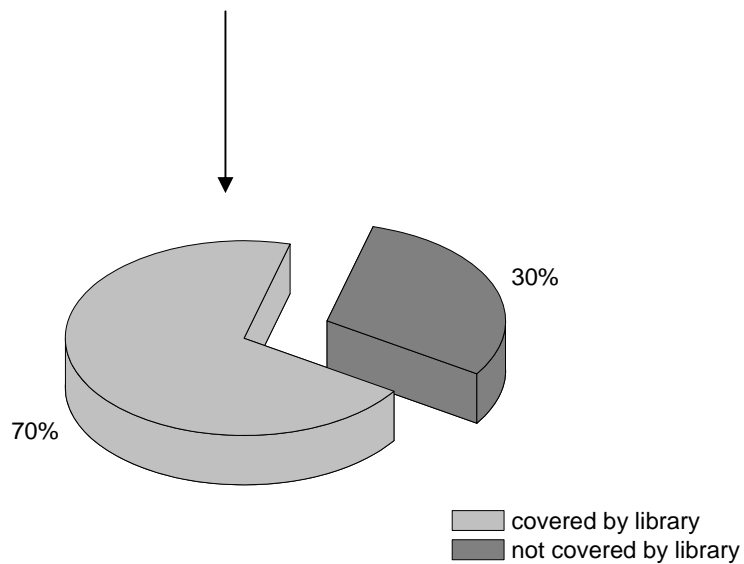

Deletion mutant library

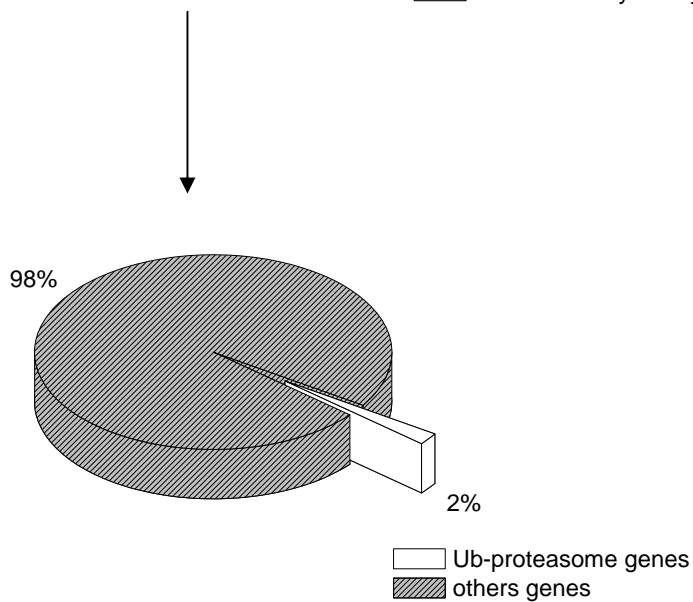

Supplement: Additional file 1 — Figure S1. Scheme of the deletion haploid mutant library. The scheme represents the deletion haploid mutant library in terms of percentage of non-essential genes and of Ub-proteasome genes as compared to the whole S. pombe genome. [file 1471-2164-12-44-S1.PDF]

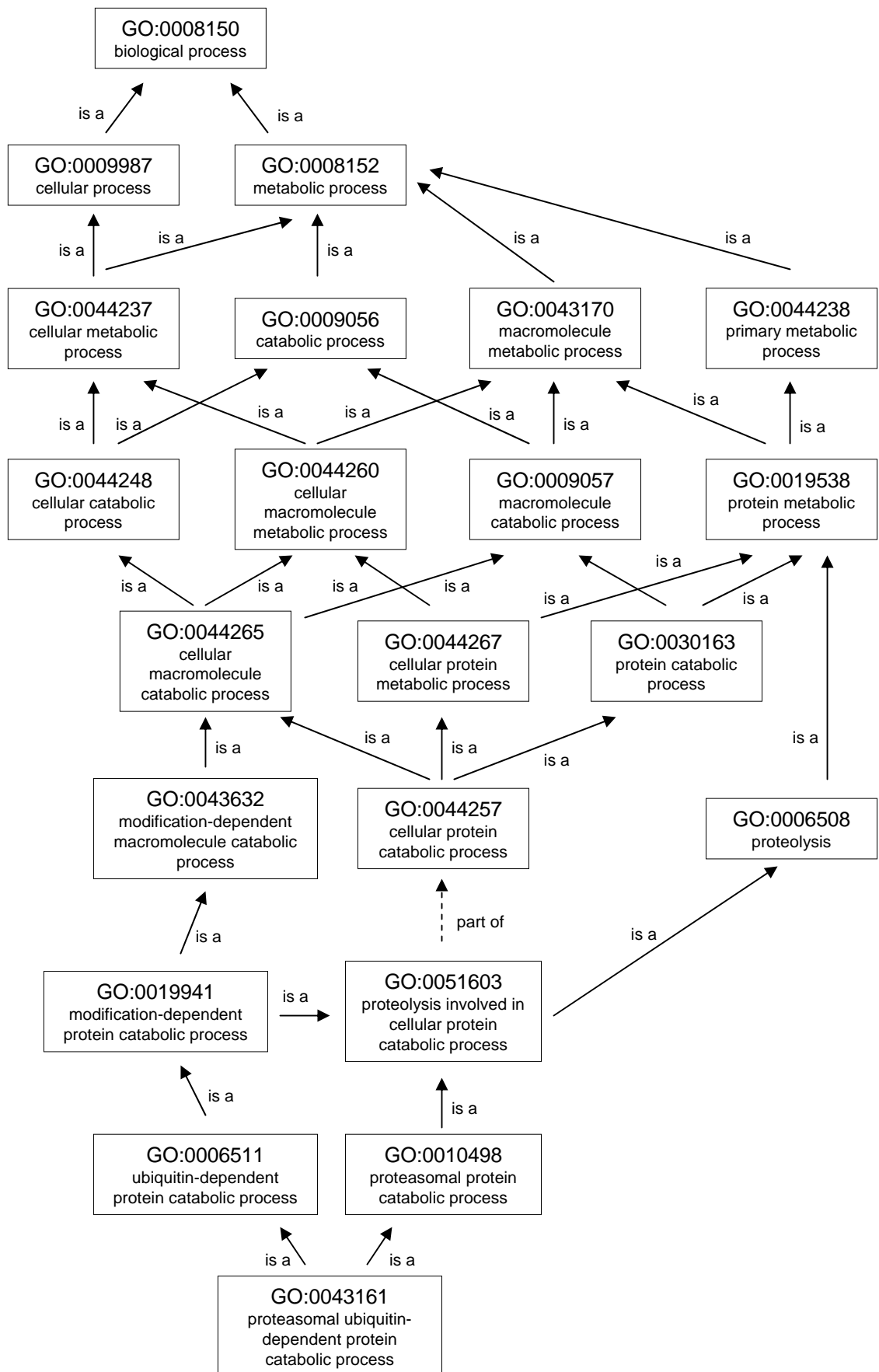

Supplement: Additional file 2 — figure S2. Tree view from The Gene Ontology http://geneontology.org relative to the Ub-proteasome system in S. pombe. In particular, the "proteasomal ubiquitin-dependent protein catabolic process (GO:0043161)" annotation is shown. The tree structure holds information about GO terms and the relationships between them. Each box in the tree view contains the GO ID and the GO term name. [file 1471-2164-12-44-S2.PDF]

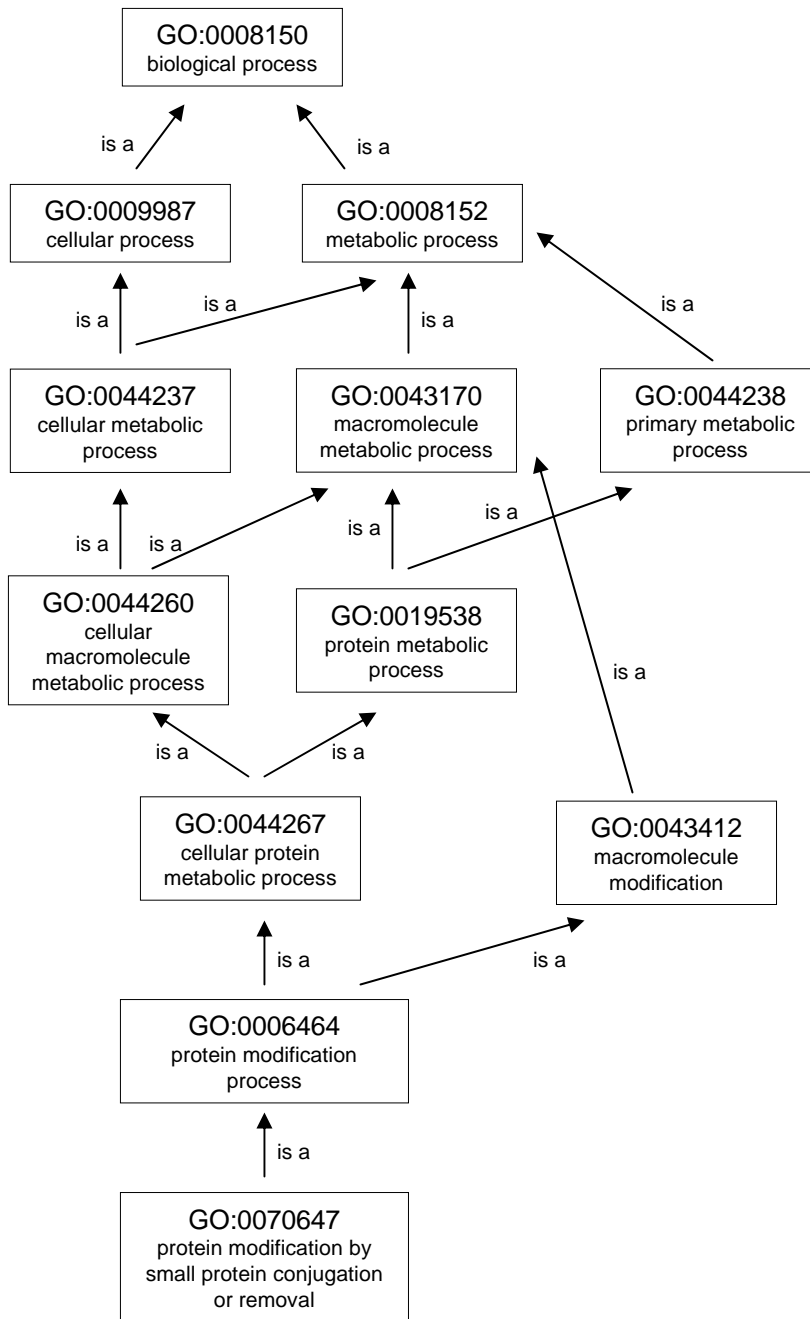

Supplement: Additional file 3 — figure S3. Tree view from The Gene Ontology http://geneontology.org relative to the Ub-proteasome system in S. pombe. In particular, the "protein modification by small protein conjugation or removal (GO:0070647)" annotation is shown. The tree structure holds information about GO terms and the relationships between them. Each box in the tree view contains the GO ID and the GO term name. [file 1471-2164-12-44-S3.PDF]
